# Age-related diagnostic value of D-dimer testing and the role of inflammation in patients with suspected deep vein thrombosis

**DOI:** 10.1038/s41598-017-04843-x

**Published:** 2017-07-04

**Authors:** Jürgen H. Prochaska, Bernd Frank, Markus Nagler, Heidrun Lamparter, Gerhard Weißer, Andreas Schulz, Lisa Eggebrecht, Sebastian Göbel, Natalie Arnold, Marina Panova-Noeva, Iris Hermanns, Antonio Pinto, Stavros Konstantinides, Hugo ten Cate, Karl J Lackner, Thomas Münzel, Christine Espinola-Klein, Philipp S. Wild

**Affiliations:** 1 Center for Cardiology – Cardiology I, University Medical Center, Johannes Gutenberg University Mainz, 55131 Mainz, Germany; 2 Center for Thrombosis and Hemostasis (CTH), University Medical Center, Johannes Gutenberg University Mainz, 55131 Mainz, Germany; 3 German Center for Cardiovascular Research (DZHK), partner site Rhine Main, 55131 Mainz, Germany; 4 Center for Translational Vascular Biology (CTVB), University Medical Center, Johannes Gutenberg University Mainz, 55131 Mainz, Germany; 5 Preventive Cardiology and Preventive Medicine, Center for Cardiology, University Medical Center, Johannes Gutenberg University Mainz, 55131 Mainz, Germany; 6 Laboratory for Clinical Thrombosis and Hemostasis, Department of Internal Medicine, Cardiovascular Research Institute Maastricht (CARIM), Maastricht University Medical Center, 6229 ER Maastricht, The Netherlands; 7 Institute of Clinical Chemistry and Laboratory Medicine, University Medical Center, Johannes Gutenberg University Mainz, 55131 Mainz, Germany

## Abstract

Previous reports have investigated the impact of age on D-Dimer testing in elderly individuals with suspected deep vein thrombosis (DVT), but data on the age-related diagnostic value of D-dimer in a sample covering a broad age range are limited. The present study determined age-specifically the diagnostic accuracy of D-dimer and compared it to C-reactive protein (CRP), a marker of inflammation, in 500 patients with suspected DVT from the VTEval project (NCT02156401). Sensitivity of D-dimer was lower in patients < 60 years in comparison to patients ≥ 60 years (∆−16.8%), whereas specificity was 27.9% higher. Lowest levels of sensitivity were detected for female sex, unprovoked DVT, low thrombotic burden, and distal DVT. A fixed D-dimer threshold of 0.25 mg/L FEU resulted in elevated sensitivity for patients < 60 with a reduction of false negatives by 40.0% for proximal DVT and by 50.0% for distal DVT. In patients < 60 years, D-dimer and CRP demonstrated comparable diagnostic performance for both proximal and distal DVT (p > 0.05). In conclusion, these data outline a clinically-relevant limitation of D-dimer testing among younger patients with suspected DVT indicating a necessity for age-adapted cut-off values. Further research is required to decrypt the role of inflammation in the pathophysiology and diagnosis of venous thrombosis.

## Introduction

Deep vein thrombosis (DVT) is a common disease with an age-dependently increasing incidence. It bears the risk for sequelae comprising pulmonary embolism as potentially life-threatening complication, post-thrombotic syndrome and recurrent venous thromboembolism^1^. A combined assessment of the clinical probability of DVT and the measurement of the circulating D-dimer concentration are currently recommended as initial diagnostic approach for patients presenting with suspected proximal DVT, followed by compression duplex ultrasound (CDUS) as gold standard^2^.

Although D-dimer is very specific for fibrin, the specificity for venous thromboembolism is poor^3^. Several clinical presentations have been demonstrated to come along with elevated concentrations of D-dimer (e.g. infection, malignancy, pregnancy)^4^. The finding that D-dimer concentrations increase with age implies practical limitations of D-dimer testing in clinical routine^5^. Therefore, age-dependent cut-off levels have been proposed for D-dimer testing in current guidelines for patients with suspected pulmonary embolism^6^. In contrast, little attention has been drawn to the effect of age on the diagnostic performance of D-dimer in patients with suspected deep vein thrombosis, especially with regard to the distinct sub-entities proximal DVT and isolated distal DVT^7^.

Against this background we aimed to assess the age-related diagnostic performance of D-dimer in patients presenting to a high-volume vascular centre for CDUS with suspected DVT. In order to display the strengths and weaknesses of D-dimer testing, we analysed its utility also with respect to distinct subtypes of DVT and compared its diagnostic performance to the inflammatory biomarker C-reactive protein (CRP).

## Results

**Figure 1:**
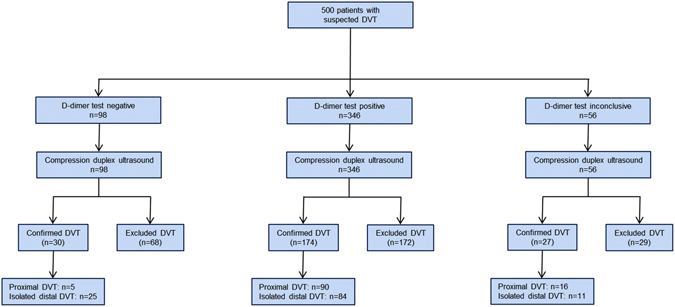
Flow diagram for a threshold of D-dimer at 0.5 mg/L FEU. Reasons for inconclusive test results were absence quality controlled data on concentration of D-Dimer in N = 56 individuals due to quantitative D-dimer testing, qualitative D-dimer test by non-certified external laboratories, or absence of adequate biomaterial. DVT, deep vein thrombosis.

The analysis comprised data of 500 patients with suspected DVT, who were enrolled between April 2013 and June 2015. The characteristics of the study sample with a median age of 60.0 (IQR 45.0/72.0) years and slightly more females are presented in Table 1. The proportion of outpatients was 87.4% (n = 437) in the sample. In total, the cohort comprised 231 patients with confirmed DVT, whereas DVT was excluded in 269 patients. Among DVT cases, diagnoses of proximal DVT (48.1%) and isolated distal DVT (51.9%) were almost equally distributed. The correlation of the two D-dimer assays, which were used for D-dimer testing during the course of the study, was 0.973 (see Supplemental Table 1 and Supplemental Fig. 1). The STARD diagram describing the flow of participants through the study is displayed in Fig. 1.

**Table 1:** Clinical characteristics of patients with suspected DVT.

	Patients with suspected DVT
Sample size, n	500
Sex (female), % (n)	55.6 (278)
Age [years]	60.0 (45.0/72.0)
Diagnosis of DVT	46.2 (231)
Proximal DVT, % (n)	22.2 (111)
Isolated distal DVT, % (n)	24.0 (120)
Aetiology of DVT	
Provoked, % (n)	26.0 (130)
Unprovoked, % (n)	19.4 (97)
Number of venous segments with thrombotic material	
1, % (n)	27.8 (139)
≥2, % (n)	18.4 (92)
Clinical signs and predisposing factors of DVT	
Active cancer, % (n)	17.0 (85)
Alternative diagnosis at least as likely as deep vein thrombosis, % (n)	12.8 (64)
Bedridden recently for ≥ 3 days or major surgery within previous 12 weeks, % (n)	13.4 (67)
Calf swelling ≥ 3 cm larger than that on the asymptomatic side, % (n)	10.2 (51)
Collateral (non-varicose) superficial veins, % (n)	5.8 (29)
Entire leg swollen, % (n)	12.0 (60)
Localized tenderness along the distribution of the deep venous system, % (n)	33.9 (169)
Paralysis, paresis or recent plaster immobilization of the lower extremities, % (n)	7.8 (39)
Pitting oedema confined to the symptomatic leg, % (n)	23.0 (115)
Previously documented DVT, % (n)	24.4 (122)
Wells score	1.2 ± 1.4
Concentration of humoral biomarkers	
D-dimer [mg/L FEU]	1.14 (0.53/2.40)
CRP [mg/L]	6.80 (2.50/20.0)

CRP, C-reactive protein; DVT, deep vein thrombosis; FEU, fibrinogen equivalent unit.

### D-dimer for the diagnosis of DVT

**Figure 2:**
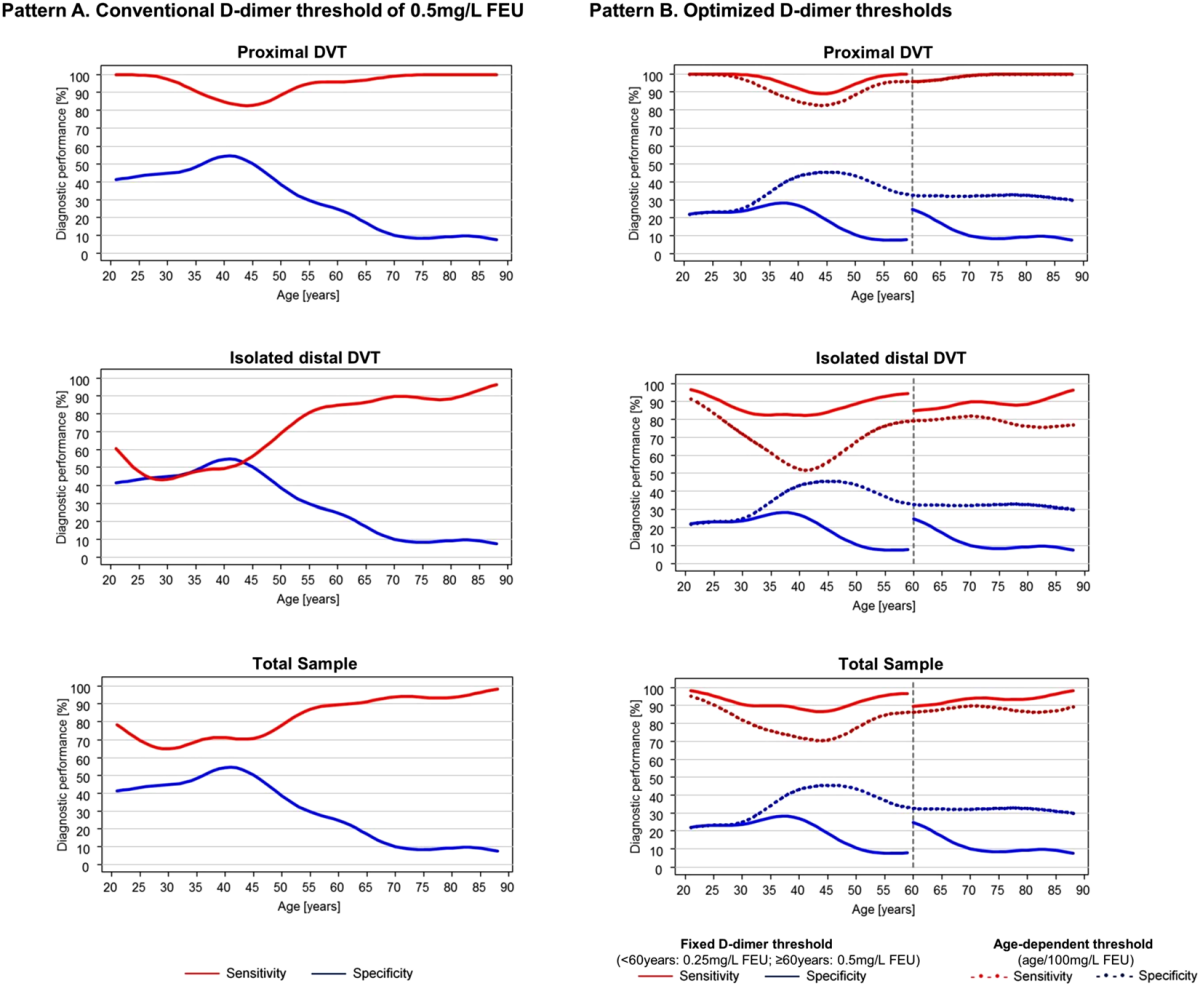
Age-dependent diagnostic performance of D-dimer in patients with suspected DVT according to different diagnostic thresholds. Pattern A (left side) illustrates the diagnostic performance of the established D-dimer threshold of 0.5 mg/L FEU. Sensitivity (red line) and specificity (blue line) of D-dimer are displayed according to age for patients with proximal DVT, patients with isolated distal DVT, and in the total sample. Pattern B (right side) depicts the diagnostic performance of optimized D-dimer thresholds for sensitivity (red line) and specificity (blue line) in proximal DVT, isolated distal DVT, and the total sample. Solid lines illustrate age-specific fixed thresholds (for patients < 60 years: 0.25 mg/L FEU D-dimer and for patients ≥ 60 years: 0.5 mg/L FEU D-dimer), whereas dashed lines show an age-dependent threshold (age/100 mg/L FEU D-dimer). DVT, deep vein thrombosis.

Age-specific levels of sensitivity and specificity of D-dimer are illustrated in Fig. 2A for patients with proximal DVT, isolated distal DVT and the total sample. As the diagnostic performance of D-dimer was worse at younger ages, the sample was stratified accordingly by the median age of the study sample, i.e. 60 years for further analysis. The distribution of clinical characteristics for both age groups is outlined in Supplemental Table 2. Comparison of the diagnostic performance of D-dimer between both age groups indicated marked differences regarding sensitivity and specificity for both the total sample and pre-specified subgroups (Table 2): In general, the sensitivity of D-dimer was always lower for the younger individuals. Intra-group analysis of patients < 60 years revealed the largest differences in sensitivity with regard to sex (female vs. male sex: −13.0%), aetiology of DVT (unprovoked vs. provoked: −13.1%), number of venous segments with thrombotic material (one segment vs. at least two segments: −36.3%), and site of DVT (isolated distal DVT vs proximal DVT: −20.7%).

**Table 2:** Diagnostic performance of D-dimer in patients with suspected DVT (N = 444) according to age groups.

		Age < 60 years					Age ≥ 60 years				
		No. DVT	Sensitivity [%]	Specificity [%]	PPV [%]	NPV [%]	No. DVT	Sensitivity [%]	Specificity [%]	PPV [%]	NPV [%]
Total sample		92/224	76.1 (66.1/84.4)	40.9 (32.4/49.8)	47.3 (39.0/55.7)	71.1 (59.5/80.9)	112/220	92.9 (86.4/96.9)	13.0 (7.3/20.08)	52.5 (45.3/59.6)	63.6 (40.7/82.8)
Sex	Male	50/95	82.0 (68.6/91.4)	37.8 (23.8/53.5)	59.4 (46.9/71.1)	65.4 (44.3/82.8)	64/105	95.3 (86.9/99.0)	17.1 (7.2/32.1)	64.2 (53.7/73.8)	70.0 (34.8/93.3)
	Female	42/129	69.0 (52.9/82.4)	42.5 (32.0/53.6)	36.7 (26.1/48.3)	74.0 (59.7/85.4)	48/115	89.6 (77.3/96.5)	10.4 (4.3/20.3)	41.7 (32.1/51.9)	58.3 (27.7/84.8)
Aetiology	Unprovoked	42/174	69.0 (52.9/82.4)	40.9 (32.4/49.8)	27.1 (19.0/36.6)	80.6 (69.1/89.2)	44/152	90.9 (78.3/97.5)	13.0 (7.3/20.8)	29.9 (22.3/38.4)	77.8 (52.4/93.6)
	Provoked	47/179	83.0 (69.2/92.4)	40.9 (32.4/49.8)	33.3 (24.9/42.6)	87.1 (76.1/94.3)	67/175	94.0 (85.4/98.3)	13.0 (7.3/20.8)	40.1 (32.4/48.2)	77.8 (52.4/93.6)
Number of segments with thrombi	1	54/186	61.1 (46.9/74.1)	40.9 (32.4/49.8)	29.7 (21.4/39.1)	72.0 (60.4/81.8)	72/180	90.3 (81.0/96.0)	13.0 (7.3/20.8)	40.9 (33.2/48.9)	66.7 (43.0/85.4)
	≥2	38/170	97.4 (86.2/99.9)	40.9 (32.4/49.8)	32.2 (23.8/41.5)	98.2 (90.3/100)	40/148	97.5 (86.8/99.9)	13.0 (7.3/20.8)	29.3 (21.8/37.8)	93.3 (68.1/99.8)
Pretest probability	Low-to-moderate	71/195	73.2 (61.4/83.1)	41.1 (32.4/50.3)	41.6 (32.9/50.8)	72.9 (60.9/82.8)	90/179	94.4 (87.5/98.2)	13.5 (7.2/22.4)	52.5 (44.5/60.4)	70.6 (44.4/89.7)
	High	21/29	85.7 (63.7/97.0)	37.5 (8.5/75.5)	78.3 (56.3/92.5)	50.0 (11.8/88.2)	22/40	86.4 (65.1/97.1)	11.1 (1.4/34.7)	54.3 (36.6/71.2)	40.0 (5.3/85.3)
Site	Proximal	46/178	91.3 (79.2/97.6)	40.9 (32.4/49.8)	35.0 (26.5/44.2)	93.1 (83.3/98.1)	49/157	98.0 (89.1/99.9)	13.0 (7.3/20.8)	33.8 (26.1/42.2)	93.3 (68.1/99.8)
	Isolated distal	46/178	60.9 (45.4/74.9)	40.9 (32.4/49.8)	26.4 (18.3/35.9)	75.0 (63.4/84.5)	63/171	88.9 (78.4/95.4)	13.0 (7.3/20.8)	37.3 (29.6/45.6)	66.7 (43.0/85.4)

Sensitivity, specificity, positive predictive value (PPV) and negative predictive value (NPV) are provided as relative frequency with according 95% confidence interval for a D-dimer threshold of 0.5 mg/L FEU. Absolute frequency (no.) of DVT cases is denoted for all subgroups. Reference group for the stratified analysis of DVT cases by aetiology, site, and number of venous segments with thrombotic material was patients without DVT. Classification of pre-test probability: low-to-moderate: Wells score 0–2; high: Wells score > 2. DVT, deep vein thrombosis.

### Subgroup analyses of D-dimer testing

#### Proximal DVT versus distal DVT

For proximal DVT, sex-specific differences in the utility of D-dimer were assessed: for patients < 60 years of age, levels of sensitivity were 10.3% lower in females as compared to males. This finding was complemented by lower levels of sensitivity in unprovoked DVT (−15.2%) and in patients with a single thrombosed venous segment (−34.9%). For all sub-categories of patients ≥ 60 years, higher levels of sensitivity where observed (92.3% to 100%), which were accompanied by lower specificity (10.6% to 17.5%; see Supplemental Table 3). For isolated distal DVT, the absolute differences of sensitivity and specificity between both age groups were considerably higher as compared to proximal DVT. The differences in sensitivity between both age groups were most pronounced for female sex (−32.0%) and unprovoked DVT (−24.7%). Specificity was approximately 30% lower in patients ≥ 60 years of age in comparison to those < 60 years (see Supplemental Table 4).

#### Low-to-moderate pretest probability

In individuals with low-to-moderate pretest probability, a marked difference in test characteristics between age groups was detected: in patients < 60 years, sensitivity was lower at low-to-moderate pretest probability (73.2%, 95% confidence interval (CI) 61.4%/83.1%) as compared to patients with a high pretest probability (85.7%, 95%CI 63.7%/97.0%). For proximal DVT, sensitivity of D-dimer in individuals under 60 years at low-to-moderate pretest probability was 87.5% (95%CI 71.0%/96.5%). In contrast, sensitivity of D-dimer at low-to-moderate pretest probability was higher in patients ≥ 60 years for the total sample of DVT (94.4%, 95%CI 87.5%/98.2%) and the corresponding subsample of proximal DVT (100%, 95%CI 85.5%/100%), respectively.

#### Outpatients

Restriction of the study sample to outpatients confirmed age-related differences for D-dimer testing: in line with the results for the total sample, sensitivity of D-dimer was 18.2% lower in outpatients < 60 years of age as compared to outpatients ≥ 60 years (74.4%, 95%CI 63.6%/83.4%, vs. 92.6%, 95%CI 85.3%/97.0%). Similarly, lower levels of sensitivity were observed for proximal and distal DVTs in outpatients < 60 years compared to the elderly (∆sensitivity −9.8% and −27.8%, see Supplemental Table 5).

### Optimized thresholds for D-dimer testing

An age-dependent threshold for D-dimer (age/100 mg/L FEU), which had recently been evaluated for elderly patients with suspected pulmonary embolism^8^, was analysed in patients with suspected DVT ≥ 60years of age: for proximal DVT, this cut-off value increased the specificity of D-dimer at steady levels of sensitivity. For isolated distal DVT, the increase in specificity was accompanied by lower levels of sensitivity compared to the established threshold of 0.5 mg/L FEU (Fig. 2B).

Due to the low level of sensitivity for D-dimer testing in younger individuals < 60 years, alternative age-specific cut-off values were evaluated for the biomarker: the age-dependent threshold (age/100 mg/L FEU) was compared to optimized fixed cut-off values, which may result in a higher feasibility in clinical routine, for both age groups. For proximal DVT, a fixed threshold of D-dimer displaying a sensitivity of 95% was detected at 0.25 mg/L FEU. In comparison to the common cut-off (0.5 mg/L FEU) and an age-dependent threshold (age/100 mg/L FEU), the fixed lower cut-off displayed higher sensitivity for patients < 60 years of age in the total sample and the subsamples of proximal and isolated distal DVT, which was accompanied by lower specificity, however (Fig. 2B). The use of the optimized D-dimer threshold results in a reduction of DVT patients with negative D-dimer tests by 40.0% (2 out of 5 patients) in proximal DVT and by 50.0% in isolated distal DVT (12 out of 24 patients; see Supplemental Fig. 2). In patients at low-to-moderate pre-test probability, the D-dimer threshold of 0.25 mg/L FEU raised sensitivity of D-dimer testing in patients < 60 years from 73.2% (95%CI 61.4%/83.1%) to 91.5% (95%CI 82.5%/96.8%) at the cost of lower specificity (16.1%, 95%CI 10.1%/23.8%). Consistent observations were made in the subsample of outpatients < 60 years of age at low-to-moderate pretest probability (sensitivity: 90.9%, 95%CI 81.3%/96.6%, specificity: 15.7%, 95%CI 9.5%/23.6%; see Supplemental Table 6) resulting in an overall NPV of 92.3% (95%CI 86.7%/96.1%).

### Value of C-reactive protein in patients with suspected DVT

**Figure 3:**
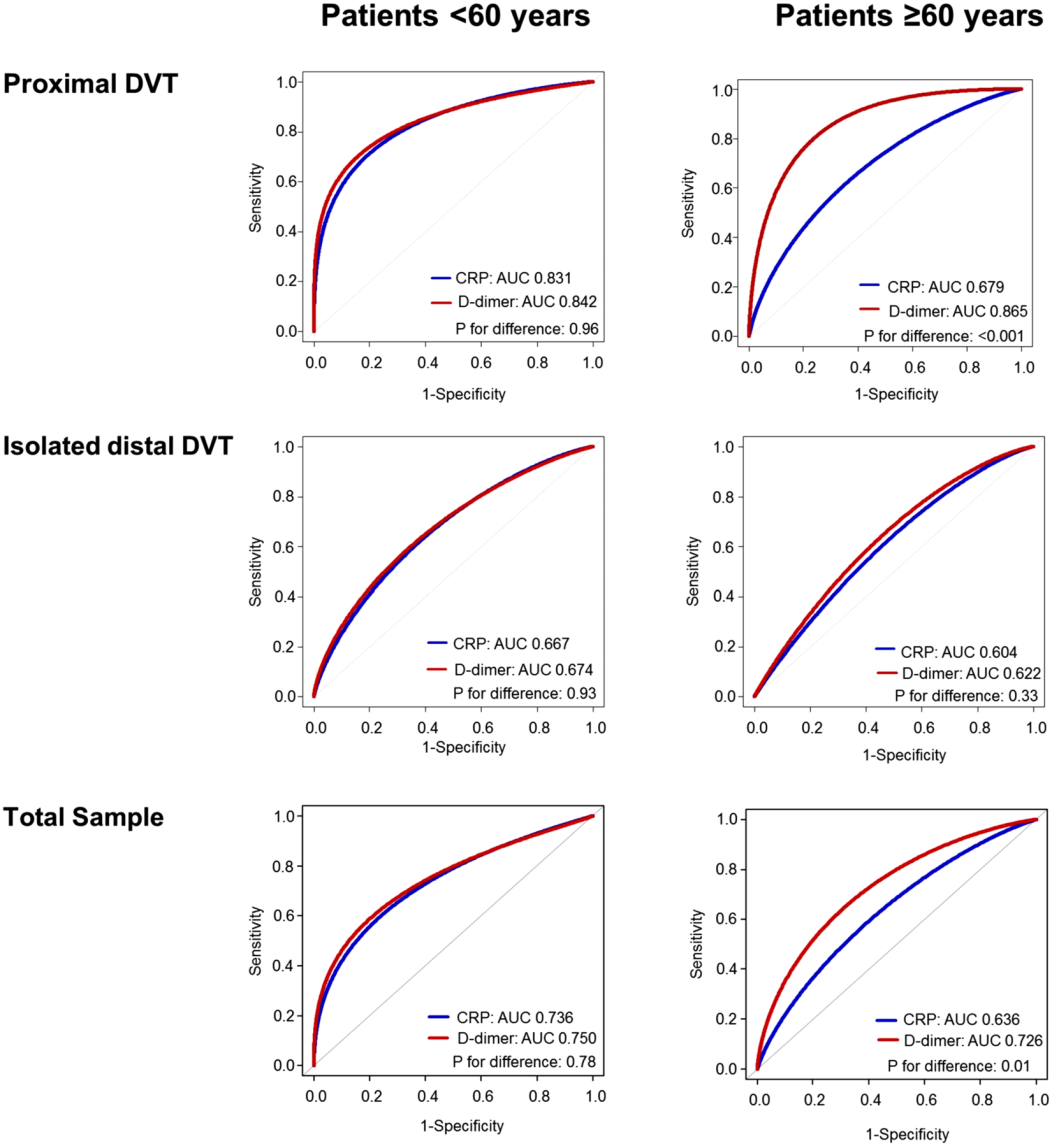
Comparison of the diagnostic performance of D-dimer and CRP in patients with suspected DVT according to age and type of DVT. ROC curves are displayed for D-dimer (red line) and CRP (blue line) stratified by age and site of DVT. P for difference is calculated for the AUC of the biomarkers in the respective analyses. AUC, area under the receiver operating curve; DVT, deep vein thrombosis.

Against the background of the limitations of D-dimer testing in young patients with suspected DVT that were identified in the analysis and the role of inflammation in venous thrombosis^9, 10^, we evaluated CRP – a commonly available inflammatory biomarker in clinical routine – as alternative biomarker in the diagnostic setting of patients with suspected DVT (see Supplemental Tables 3, 4 and 7). After incorporation of information on sex and the Wells score, receiver operating characteristic (ROC) curves demonstrated comparable diagnostic performance of CRP and D-dimer in patients < 60 years of age for proximal DVT (AUC_D-dimer_: 0.842 vs. AUC_CRP_: 0.831) and isolated distal DVT (AUC_D-dimer_: 0.674 vs. AUC_CRP_: 0.667) (for both, *P* for difference > 0.05; Fig. 3). In patients ≥ 60 years, D-dimer showed a higher AUC in comparison to CRP for proximal DVT (AUC_D-dimer_: 0.865 vs. AUC_CRP_: 0.679; *P* for difference < .001), whereas no statistically-significant difference was detected for isolated distal DVT (AUC_D-dimer_: 0.622 vs. AUC_CRP_: 0.604; *P* for difference = 0.33).

In order to evaluate both biomarkers at best performance with regard to sensitivity, we subsequently investigated the diagnostic utility of an optimized diagnostic threshold of CRP for patients < 60. Similarly to D-dimer, a cut-off value of CRP providing 95% sensitivity for proximal DVT was identified at 1.0 mg/L. Comparison of the diagnostic performance for the optimized fixed thresholds of both biomarkers in patients < 60 years did not result in relevant differences of the diagnostic utility of CRP for proximal DVT (c-index_D-dimer_: 0.562 vs. c-index_CRP_: 0.543) or isolated distal DVT (c-index_D-dimer_: 0.518 vs. c-index_CRP_: 0.487) in comparison to D-dimer (Table 3; for both: *P* for difference > 0.05).

**Table 3:** Comparison of the diagnostic performance of optimized thresholds of D-dimer and CRP in patients with suspected DVT.

	Proximal DVT	Isolated distal DVT	Total Sample
**Patients ≥60 years**			
**Age-dependent D-dimer cut-off (age/100 mg/L FEU)**			
Sensitivity [%] (95%CI)	98.0 (89.1/99.9)	81.0 (69.1/89.8)	88.4 (81.0/93.7)
Specificity [%] (95%CI)	33.3 (24.6/43.1)	33.3 (24.6/43.1)	33.3 (24.6/43.1)
C-index	0.656	0.571	0.609
**Patients <60years**			
**Optimized D-dimer threshold (0.25 mg/L FEU)**			
Sensitivity [%] (95%CI)	96.7 (90.7/99.3)	88.8 (81.2/94.1)	92.4 (87.8/95.7)
Specificity [%] (95%CI)	15.1 (10.8/20.3)	15.1 (10.8/20.3)	15.1 (10.8/20.3)
C-index	0.560	0.518	0.538
**Optimized CRP threshold (1.0 mg/L)**			
Sensitivity [%] (95%CI)	95.6 (84.9/99.5)	84.4 (70.5/93.5)	90.0 (81.9/95.3)
Specificity [%] (95%CI)	13.0 (7.7/20.0)	13.0 (7.7/20.0)	13.0 (7.7/20.0)
C-index	0.543	0.487	0.515
P for difference in C-indexes (D-dimer vs. CRP)	0.67	0.49	0.51

The diagnostic performance of D-dimer and CRP are illustrated for optimized, age-specific diagnostic thresholds. For patients ≥ 60 years with suspected, the diagnostic performance of an age-dependent threshold (age/100 mg/L FEU D-dimer) is depicted, whereas for patients < 60 years the test characteristics of optimized fixed thresholds of D-dimer (0.25 mg/L FEU) and CRP (1.0 mg/L FEU) are compared. The absolute value of sensitivity, specificity, and C-index are displayed. *P* for difference was calculated for C-index of D-dimer with adjusted cut-off (0.25 mg/L FEU) and CRP with adjusted cut-off (1.0 mg/L). CI, confidence interval; DVT, deep vein thrombosis; FEU, fibrinogen equivalent unit.

## Discussion

The present work investigated the age-dependent performance of D-dimer testing in an all-comer study of individuals with suspected DVT and revealed clinically-relevant limitations: The diagnostic ability of D-dimer was substantially lower in individuals aged less than 60 years, especially in individuals of female sex and with unprovoked aetiology. The use of a lower fixed threshold for D-dimer (0.25 mg/L FEU) in patients < 60 years resulted in an improvement of sensitivity for both proximal DVT and isolated distal DVT at lower specificity. Of clinical importance, the discriminative ability of D-dimer and CRP did not differ for individuals < 60 years.

D-dimer assays as sensitive and non-specific markers for DVT have been studied extensively in patients with suspected DVT^11^. Sensitivity (85–98%) and specificity (26–58%) of D-dimer assays for proximal DVT vary between latex agglutination assays and whole blood assays in literature^12–14^. In the current study, D-dimer showed comparable levels of sensitivity (94.5%, 95%CI 87.6 to 98.2%) and specificity (28.6%, 95%CI 22.9 to 34.8%) for proximal DVT. The well-known increase of circulating D-dimer in patients with increasing age led to efforts evaluating the use of age-dependent thresholds of D-dimer in older patients with suspected DVT^15, 16^. In line with a previous study investigating patients > 50 years with suspected pulmonary embolism, we confirmed an increase of specificity at steady levels of sensitivity for an age-dependent D-dimer cut-off value in patients ≥ 60 years with suspected DVT^8^. However, the age-specific analysis identified decreased levels of sensitivity of D-dimer in patients < 60 years as compared to ≥ 60 years, especially in individuals with unprovoked events and with female sex, which have not been reported in literature yet. This finding was also confirmed when restricting the study sample to outpatients. Since ageing is a well-known “risk factor” to promote inflammatory processes, to alter immune response and to change the balance of primary and secondary haemostasis, both the development and process of thrombus resolution may differ between DVT patients by age^17–20^. In a mouse model of stasis induced thrombosis, aging was demonstrated to be associated with impaired thrombus resolution^20^. Interestingly, investigations in paediatric samples lead to the concept of developmental haemostasis indicating that the levels of markers reflecting the coagulation system already differ at early stage in life^21^. Recently, two studies indicated a need for age-specific reference values of D-dimer due to substantially lower concentrations of D-dimer in younger healthy individuals as compared to the elderly^22, 23^. In the present study, the use of a fixed threshold of 0.25 mg/L FEU for D-dimer in patients < 60 years resulted in an improvement of sensitivity for both proximal and isolated distal DVT. This improvement of sensitivity was especially observed in the clinically-relevant group of patients with suspected DVT at low-to-moderate pre-test probability. As expected, the approach to decrease the amount of false negatives (i.e. missed cases of DVT) was accompanied by lower specificity as cut-off values represent always a trade-off between sensitivity and specificity^24^. Therefore, age-range specific cut-off values of D-dimer merit consideration for the clinical diagnostic setting of DVT.

It is known from literature that D-dimer has lower sensitivity in isolated distal than in proximal DVT. The lower sensitivity in patients with isolated distal DVT in the present study compared to a recent report is explained by the higher frequency of young individuals, which implies lower levels of sensitivity^25^. The low sensitivity of D-dimer in isolated distal DVT substantiates the dependence of the diagnostic accuracy of this marker on the extent of thrombotic burden or lower fibrinolytic activity, coherently to the correlation of its diagnostic accuracy with the number of segments with thrombotic material in patients with proximal DVT. The different diagnostic ability of D-dimer testing in patients with proximal and isolated distal DVT merits careful attention since D-dimer testing, which is routinely available in outpatient settings via quantitative and qualitative D-dimer assays, is commonly applied to patients with suspected DVT < 60 years irrespective of the DVT phenotype^26^. The present study showed a sensitivity of 87.0% for isolated distal DVT at an optimized D-dimer threshold of 0.25 mg/L FEU for patients < 60 years, although it is still to be critically evaluated whether a specificity of <20% is acceptable for routine application in daily practice.

The finding that CRP was non-inferior to D-dimer for diagnostics in all subgroups of younger patients with suspected DVT sheds new light on the role of inflammatory processes in the pathophysiology of DVT. CRP reflects systemic inflammation as acute-phase protein and is often routinely measured in patients with elevated levels of D-dimer in the absence of clinical symptoms of DVT. Although preclinical studies have established that DVT and inflammatory processes are interacting, scientific evidence has been conflicting regarding the diagnostic utility of CRP as biomarker in patients with suspected DVT^27–29^. The present study indicates that CRP can be considered as an alternative marker to D-dimer in patients with suspected DVT aged <60 years. The variability in the inflammatory and immune response dependent on age – “inflammaging” and “immunosenescence” – explains the differences observed in the diagnostic performance of D-dimer and the presence of DVT^30, 31^. The concept of immunothrombosis should be considered, which refers to the evolutionary conserved link between coagulation and innate immunity, and focusses on the prothrombotic ability of innate immune cells^10^. Last, since elevated levels of CRP are frequently observed in health states, which coincide with increasing age (e.g. diabetes, cardiovascular disease, cancer or rheumatic disorders), it seems consistent that CRP shows a better diagnostic performance in younger individuals, who are more likely to have lower levels in a DVT-free status, as compared to the elderly^32, 33^.

### Limitations

There are several limitations of the present study, which merit consideration. The lower levels of sensitivity in individuals with female sex and unprovoked DVT underline the need for optimization of diagnostic approaches and risk assessment strategies^34, 35^. However, the sample size – despite the fairly large size compared to literature – restricted the assessment of sex-specific cut-off values and subgroup analyses. Moreover, two assays for D-dimer testing have been applied in the present study, which might have introduced a bias that contributes to the explanation of the findings. Yet, it seems unlikely, since the assays show a high correlation (r = 0.973, see lemental Table 1 and Supplemental Fig. 1). Finally, when interpreting this study, it needs to be considered that the performance of clinical decision rules such as the Well score, and the prevalence of DVT cases as well as the number of false positives and false negatives vary across settings^36, 37^. Therefore, extrapolation of the results to other health-care settings (e.g. non-academic centres) or populations (e.g. with different ethnicity) should be done with caution. The reproducibility of the results of this observational diagnostic study needs to be validated in other cohorts and merits further investigation by future studies (e.g. outcome study for the ascertainment of clinical relevance).

## Conclusion

This study demonstrates a substantially lower sensitivity of D-dimer in individuals with suspected DVT aged less than 60 years. In this age group, especially individuals with female sex, unprovoked DVT and low thrombotic burden of DVT were identified as risk groups for worse diagnostic abilities of the marker. An optimized age-specific D-dimer threshold was defined with 0.25 mg/L FEU for these patients resulting in a substantial improvement of sensitivity, especially in patients at low-to-moderate pre-test probability. Of clinical importance, the diagnostic performance of CRP in suspected DVT was comparable to that of D-dimer in all subgroups of patients < 60 years of age in the study sample.

## Methods

### Study design

The background, rationale and study design of the VTEval project have been published recently^38^. The study is carried out by the Center for Thrombosis and Hemostasis in Mid-Western Germany, recruiting patients with a clinical suspicion of acute DVT aged ≥ 18 years. All study participants provided informed written consent for the study participation. The study conduct is performed according to the principles of good clinical practice and the principles outlined in the Declaration of Helsinki; approval by the local ethics committee (medical association of the federal state Rhineland-Palatinate, Germany; reference no. 837.320.12 (8421-F)) and the data safety commissioner of the University Medical Center of the Johannes Gutenberg University Mainz was obtained. The VTEval project was registered online at clinicaltrials.gov (identifier: NCT02156401).

### Assessment of clinical phenotype

The sample for the present report represents a consecutive sample of patients with suspected DVT, who were examined in the Department of Angiology at the University Medical Center Mainz between April 2013 and July 2015. Within the investigation, a standardized clinical examination, a computer-assisted personal interview and venous blood sampling were performed according to standard operating procedures. Blood samples were collected from all participants before conduct of CDUS. All patients with suspected DVT underwent a standardized clinical examination considering signs and symptoms of acute DVT. Subsequently, Wells score was calculated and the cohort was divided according to pre-test probability into subgroups. All study participants underwent CDUS after assessment of Wells score and measurement D-dimer concentration. All information was entered in electronic case report forms with pre-defined validity checks and cross-checked with clinical records. All study information including clinical and laboratory data underwent data management with detailed quality control and checks for plausibility.

### Measurement of biomarkers and diagnosis of DVT

Concentrations of D-dimer and CRP were collected in citrated venous blood samples. D-dimer was quantified by quantitative turbidimetric immunoassays: Innovance D-dimer BCS (Siemens Healthcare Diagnostics Products, Germany) from 04/2013 to 07/2014 and HemosIL D-dimer HS 500 for ACL TOP 700 (Instrumentation Laboratory, USA) from 08/2014 to the end of study. C-reactive protein was measured by a latex particle-enhanced turbidimetric immunoassay (Abbott Laboratories, USA). Biomarkers were categorized as within or above normal range according to the clinically established cut-off values of 0.5 mg/L fibrinogen equivalent unit (FEU) for D-dimer and 5.0 mg/L for CRP.

Participants were diagnosed as DVT cases by trained angiologists, who were blinded from the Wells score and D-dimer results, based upon the results of CDUS. Clinical decision on diagnosis or exclusion of DVT was validated by an experienced, board-certified senior angiologist. Proximal DVT was defined as venous thrombosis in the popliteal, femoral, or iliac vein of the lower limb, whereas isolated distal DVT was defined as being located below the knee with confinement to the calf veins (peroneal, posterior, anterior tibial, and muscular veins). In the case of DVT, patients received oral anticoagulation therapy according to current guidelines. Based on the medical report and the information obtained during the investigation, cases were subsequently categorized into subgroups (unprovoked vs. provoked DVT, proximal versus isolated distal DVT).

### Statistical analysis

Dichotomous variables are presented as absolute and relative frequencies. Continuous variables are reported as mean with standard deviation (SD) for normally distributed values or as median with interquartile range (IQR) where appropriate. Test characteristics (sensitivity, specificity, positive predictive value (PPV) and negative predictive values (NPV)) were calculated. Receiver operating characteristic curves were built plotting sensitivity vs. 1-specificity and calculating the area under curve (AUC). The age-dependent diagnostic performance of D-dimer was illustrated graphically by calculation of sensitivity and specificity in moving age regions with anti-proportional weighting. The manuscript was written under consideration of the Standards for Reporting Diagnostic accuracy studies^39^. All statistical analyses were conducted in R (version 3.2.2)^40^.

## Electronic supplementary material

supplemental appendix
