# Supplementary material for: Age-related diagnostic value of D-dimer testing and the role of inflammation in patients with suspected deep vein thrombosis
Source: Sci Rep. 2017 Jul 4;7:4591. doi: 10.1038/s41598-017-04843-x (PMC5496875; doi:10.1038/s41598-017-04843-x)
Supplement: Supplementary file 1 — supplemental appendix [file 41598_2017_4843_MOESM1_ESM.doc]

**SUPPLEMENTAL APPENDIX**

**Age-related diagnostic value of D-dimer testing and the role of inflammation in patients with suspected deep vein thrombosis**

Running head: Age-related diagnostic value of D-dimer in DVT

Jürgen H. Prochaska^1,2,3,4,*^, Bernd Frank^2,4,*^, Markus Nagler^2,4^, Heidrun Lamparter^2,4^, Gerhard Weißer^1,4^, Andreas Schulz^5,4^, Lisa Eggebrecht^5,4^, Sebastian Göbel^1,3,4^, Natalie Arnold^5,4^, Marina Panova-Noeva^2,4^, Iris Hermanns^5,4^, Antonio Pinto^5,4^, Stavros Konstantinides^2^, Hugo ten Cate^6,2^, Karl J Lackner^7,3,4^, Thomas Münzel^1,3,2,4^, Christine Espinola-Klein^1,4^, Philipp S. Wild^5,2,3,4^

^1^ Center for Cardiology – Cardiology I, University Medical Center, Johannes Gutenberg University Mainz, 55131 Mainz, Germany

^2^ Center for Thrombosis and Hemostasis (CTH), University Medical Center, Johannes Gutenberg University Mainz, 55131 Mainz, Germany

^3^ German Center for Cardiovascular Research (DZHK), partner site Rhine Main, 55131 Mainz, Germany

^4^ Center for Translational Vascular Biology (CTVB), University Medical Center, Johannes Gutenberg University Mainz, 55131 Mainz, Germany

^5^ Preventive Cardiology and Preventive Medicine, Center for Cardiology, University Medical Center, Johannes Gutenberg University Mainz, 55131 Mainz, Germany

^6^ Laboratory for Clinical Thrombosis and Hemostasis, Department of Internal Medicine, Cardiovascular Research Institute Maastricht (CARIM), Maastricht University Medical Center, 6229 ER, Maastricht, the Netherlands

^7^ Institute of Clinical Chemistry and Laboratory Medicine, University Medical Center, Johannes Gutenberg University Mainz, 55131 Mainz, Germany

^*^ JHP and BF contributed equally and should be considered as first authors.

**Address for correspondence:**

Philipp Sebastian Wild, MD, MSc

Professor of Clinical Epidemiology

University Medical Center of the Johannes Gutenberg-University Mainz

Langenbeckstr. 1, 55131 Mainz, Germany

Phone: +49 6131 17-7163

Fax: +49 6131 17-8640

Email: philipp.wild@unimedizin-mainz.de

**Table of contents**

**Page**

Table S1………………………………………………………………………………...….…………………….3

Table S2…………………………………………………………………………………………….……………4

Table S3……………………………………………………………………………….…………………………5

Table S4………………………………………………………………….………………………………………6

Table S5…………………………………………………….……………………………………………………7

Table S6…………………………………………………….……………………………………………………8

Table S7…………………………………………………….……………………………………………………9

Figure S1.……………………………………….……………………………………………..........................10

Figure S2………………………….…………………………………………………………………………....11

**Table S1. Comparison of D-dimer assays.**

|  | **Siemens Innovance BCS** | **HemosIL ACL TOP** |
| --- | --- | --- |
| Sample size | 50 | 50 |
| Mean D-dimer [mg/l FEU] | 2.390 | 2.669 |
| Median D-dimer [mg/l FEU] | 1.045 | 1.191 |
| Minimum D-dimer [mg/l FEU] | 0.170 | 0.213 |
| Maximum D-dimer [mg/l FEU] | 12.440 | 15.484 |
| Range | 12.270 | 15.271 |

**Table S2. Characteristics of patients with suspected DVT according to age groups.**

|  | **Patients with suspected DVT**  **<60 years of age** | **Patients with suspected DVT**  **≥60 years of age** |
| --- | --- | --- |
| Sample size, n | 249 | 251 |
| Sex (female), % (n) | 58.2 (145) | 53.0 (133) |
| Age [years] | 45.0 (34.0/53.0) | 72.0 (65.0/76.0) |
| Diagnosis of DVT |  |  |
| Proximal DVT, % (n) | 20.9 (52) | 23.5 (59) |
| Isolated distal DVT, % (n) | 20.5 (51) | 27.5 (69) |
| Aetiology of DVT |  |  |
| Provoked, % (n) | 18.1 (45) | 29.9 (75) |
| Unprovoked, % (n) | 22.1 (55) | 20.7 (52) |
| Number of venous segments with thrombotic material |  |  |
| 1, % (n) | 23.7 (59) | 31.9 (80) |
| ≥2, % (n) | 17.7 (44) | 19.1 (48) |
| Clinical signs and predisposing factors of DVT |  |  |
| Active cancer, % (n) | 11.2 (28) | 22.7 (57) |
| Alternative diagnosis at least as likely as deep vein thrombosis, % (n) | 12.9 (32) | 12.7 (32) |
| Bedridden recently for ≥3 days or major surgery within previous 12 weeks, % (n) | 10.0 (25) | 16.7 (42) |
| Calf swelling ≥3 cm larger than that on the asymptomatic side, % (n) | 10.8 (27) | 9.6 (24) |
| Collateral (nonvaricose) superficial veins, % (n) | 6.0 (15) | 5.6 (14) |
| Entire leg swollen, % (n) | 13.7 (34) | 10.4 (26) |
| Localized tenderness along the distribution of the deep venous system, % (n) | 34.5 (86) | 33.2 (83) |
| Paralysis, paresis or recent plaster immobilization of the lower extremities, % (n) | 6.8 (17) | 8.8 (22) |
| Pitting oedema confined to the symptomatic leg, % (n) | 17.7 (44) | 28.4 (71) |
| Previously documented DVT, % (n) | 20.1 (50) | 28.7 (72) |
| Wells score | 1.1±1.4 | 1.4±1.4 |
| Concentration of humoral biomarkers |  |  |
| D-dimer [mg/L FEU] | 0.80 (0.39/1.83) | 1.46 (0.75/2.93) |
| CRP [mg/L] | 5.40 (1.90/18.08) | 8.20 (3.30/23.00) |

CRP, C-reactive protein; DVT, deep vein thrombosis; FEU, fibrinogen equivalent unit.

**Table S3. Diagnostic performance of D-dimer and CRP in patients with suspected proximal DVT.**

|  |  | **Age <60 years** | | | | |  | **Age ≥60 years** | | | | |
| --- | --- | --- | --- | --- | --- | --- | --- | --- | --- | --- | --- | --- |
|  |  | No.  DVT | Sens. [%] (95%CI) | Spec. [%]  (95%CI) | PPV [%]  (95%CI) | NPV [%]  (95%CI) |  | No.  DVT | Sens. [%] (95%CI) | Spec. [%]  (95%CI) | PPV [%]  (95%CI) | NPV [%]  (95%CI) |
| **A. D-dimer** | | | | | | | | | | | | |
| Sex | Male | 25/70 | 96.0 (79.6/99.9) | 37.8 (23.8/53.5) | 46.2 (32.2/60.5) | 94.4 (72.7/99.9) |  | 33/74 | 97.0 (84.2/99.9) | 17.1 (7.2/32.1) | 48.5 (36.0/61.1) | 87.5 (47.3/99.7) |
|  | Female | 21/108 | 85.7 (63.7/97.0) | 42.5 (32.0/53.6) | 26.5 (16.5/38.6) | 92.5 (79.6/98.4) |  | 16/83 | 100 (71.3/100) | 10.4 (4.3/20.3) | 21.1 (12.5/31.9) | 100 (47.3/100) |
| Aetiology* | Unprovoked | 16/148 | 81.2 (54.4/96.0) | 40.9 (32.4/49.8) | 14.3 (7.8/23.2) | 94.7 (85.4/98.9) |  | 15/123 | 100 (69.8/100) | 13.0 (7.3/20.8) | 13.8 (7.9/21.7) | 100 (68.1/100) |
|  | Provoked | 28/160 | 96.4 (81.7/99.9) | 40.9 (32.4/49.8) | 25.7 (17.7/35.2) | 98.2 (90.3/100) |  | 34/142 | 97.1 (84.7/99.9) | 13.0 (7.3/20.8) | 26.0 (18.6/34.5) | 93.3 (68.1/99.8) |
| Number of segments with thrombi* | 1 | 8/140 | 62.5 (24.5/91.5) | 40.9 (32.4/49.8) | 6.0 (2.0/13.5) | 94.7 (85.4/98.9) |  | 9/117 | 100 (55.5/100) | 13.0 (7.3/20.8) | 8.7 (4.1/15.9) | 100 (68.1/100) |
|  | ≥2 | 38/170 | 97.4 (86.2/99.9) | 40.9 (32.4/49.8) | 32.2 (23.8/41.5) | 98.2 (90.3/100) |  | 40/148 | 97.5 (86.8/99.9) | 13.0 (7.3/20.8) | 29.3 (21.8/37.8) | 93.3 (68.1/99.8) |
|  |  |  |  |  |  |  |  |  |  |  |  |  |
| **B. C-reactive protein** | | | | | | | | | | | | |
| Sex | Male | 25/74 | 92.0 (74.0/99.0) | 61.2 (46.2/74.8) | 54.8 (38.7/70.2) | 93.8 (79.2/99.2) |  | 32/74 | 81.2 (63.6/92.8) | 33.3 (19.6/49.5) | 48.1 (34.3/62.2) | 70.0 (45.7/88.1) |
|  | Female | 21/103 | 81.0 (58.1/94.6) | 58.5 (47.1/69.3) | 33.3 (20.8/47.9) | 92.3 (81.5/97.9) |  | 17/84 | 70.6 (44.0/89.7) | 41.8 (29.8/54.5) | 21.1 (12.5/31.9) | 84.8 (68.1/94.9) |
| Aetiology* | Unprovoked | 17/148 | 76.5 (50.1/93.2) | 59.5 (50.6/68.0) | 19.7 (10.9/31.3) | 95.1 (88.0/98.7) |  | 17/126 | 76.5 (50.1/93.2) | 38.5 (29.4/48.3) | 16.2 (8.9/26.2) | 91.3 (79.2/97.6) |
|  | Provoked | 27/158 | 92.6 (75.7/99.1) | 59.5 (50.6/68.0) | 32.1 (21.9/43.6) | 97.5 (91.3/99.7) |  | 32/141 | 78.1 (60.0/90.7) | 38.5 (29.4/48.3) | 27.2 (18.4/37.4) | 85.7 (72.8/94.1) |
| Number of segments with thrombi* | 1 | 7/138 | 42.9 (9.9/81.6) | 59.5 (50.6/68.0) | 5.4 (1.1/14.9) | 95.1 (88.0/98.7) |  | 8/117 | 75.0 (34.9/96.8) | 38.5 (29.4/48.3) | 8.2 (3.1/17.0) | 95.5 (84.5/99.4) |
|  | ≥2 | 39/170 | 94.9 (82.7/99.4) | 59.5 (50.6/68.0) | 41.1 (30.8/52.0) | 97.5 (91.3/99.7) |  | 41/150 | 78.0 (62.4/89.4) | 38.5 (29.4/48.3) | 32.3 (23.3/42.5) | 82.4 (69.1/91.6) |

Sensitivity, specificity, negative predictive value and positive predictive value are presented as percentages according to subgroups. *reference group for the stratified analysis of DVT cases by aetiology and number of venous segments with thrombotic material was patients without DVT. PPV, positive predictive value; NPV, negative predictive value; CI, confidence interval; DVT, deep vein thrombosis.

**Table S4. Diagnostic performance of D-dimer and CRP in patients with suspected isolated distal DVT.**

|  |  | **Age <60 years** | | | | |  | **Age ≥60 years** | | | | |
| --- | --- | --- | --- | --- | --- | --- | --- | --- | --- | --- | --- | --- |
|  |  | No.  DVT | Sens. [%]  (95%CI) | Spec. [%]  (95%CI) | PPV [%]  (95%CI) | NPV [%]  (95%CI) |  | No.  DVT | Sens. [%]  (95%CI) | Spec. [%]  (95%CI) | PPV [%]  (95%CI) | NPV [%]  (95%CI) |
| **A. D-dimer** | | | | | | | | | | | | |
| Sex | Male | 25/70 | 68.0 (46.5/85.1) | 37.8 (23.8/53.5) | 37.8 (23.8/53.5) | 68.0 (46.5/85.1) |  | 31/72 | 93.5 (78.6/99.2) | 17.1 (7.2/32.1) | 46.0 (33.4/59.1) | 77.8 (40.0/97.2) |
|  | Female | 21/108 | 52.4 (29.8/74.3) | 42.5 (32.0/53.6) | 18.0 (9.4/30.0) | 78.7 (64.3/89.3) |  | 32/99 | 84.4 (67.2/94.7) | 10.4 (4.3/20.3) | 31.0 (21.5/41.9) | 58.3 (27.7/84.8) |
| Aetiology* | Unprovoked | 26/158 | 61.5 (40.6/79.8) | 40.9 (32.4/49.8) | 17.0 (10.1/26.2) | 84.4 (73.1/92.2) |  | 29/137 | 86.2 (68.3/96.1) | 13.0  (7.3/20.8) | 21.0 (14.1/29.4) | 77.8 (52.4/93.6) |
|  | Provoked | 19/151 | 63.2 (38.4/83.7) | 40.9 (32.4/49.8) | 13.3 (7.1/22.1) | 88.5 (77.8/95.3) |  | 33/141 | 90.9 (75.7/98.1) | 13.0  (7.3/20.8) | 24.2 (17.0/32.7) | 82.4 (56.6/96.2) |
| **B. C-reactive protein** | | | | | | | | | | | | |
| Sex | Male | 25/74 | 48.0 (27.8/68.7) | 61.2 (46.2/74.8) | 38.7 (21.8/57.8) | 69.8 (53.9/82.8) |  | 30/72 | 60.0 (40.6/77.3) | 33.3 (19.6/49.5) | 39.1 (25.1/54.6) | 53.8 (33.4/73.4) |
|  | Female | 20/102 | 45.0 (23.1/68.5) | 58.5 (47.1/69.3) | 20.9 (10.0/36.0) | 81.4 (69.1/90.3) |  | 33/100 | 66.7 (48.2/82.0) | 41.8 (29.8/54.5) | 36.1 (24.2/49.4) | 71.8 (55.1/85.0) |
| Aetiology* | Unprovoked | 26/157 | 50.0 (29.9/70.1) | 59.5 (50.6/68.0) | 19.7 (10.9/31.3) | 85.7 (76.8/92.2) |  | 28/137 | 57.1 (37.2/75.5) | 38.5 (29.4/48.3) | 19.3 (11.4/29.4) | 77.8 (64.4/88.0) |
|  | Provoked | 18/149 | 44.4 (21.5/69.2) | 59.5 (50.6/68.0) | 13.1 (5.8/24.2) | 88.6 (80.1/94.4) |  | 34/143 | 70.6 (52.5/84.9) | 38.5 (29.4/48.3) | 26.4 (17.7/36.7) | 80.8 (67.5/90.4) |

Sensitivity, specificity, negative predictive value and positive predictive value are presented as percentages according to subgroups. *reference group for the stratified analysis of DVT cases by aetiology and number of venous segments with thrombotic material was patients without DVT. PPV, positive predictive value; NPV, negative predictive value; CI, confidence interval; DVT, deep vein thrombosis.

**Table S5. Diagnostic performance of D-dimer in outpatients with suspected DVT according to age groups.**

|  |  | **Age <60 years** | | | | |  | **Age ≥60 years** | | | | |
| --- | --- | --- | --- | --- | --- | --- | --- | --- | --- | --- | --- | --- |
|  |  | **No.**  **DVT** | **Sensitivity**  **[%]** | **Specificity**  **[%]** | **PPV**  **[%]** | **NPV**  **[%]** |  | **No.**  **DVT** | **Sensitivity**  **[%]** | **Specificity**  **[%]** | **PPV**  **[%]** | **NPV**  **[%]** |
| Total sample |  | 82/204 | 74.4 (63.6/83.4) | 42.6 (33.7/51.9) | 46.6 (37.8/55.5) | 71.2 (59.4/81.2) |  | 94/189 | 92.6 (85.3/97.0) | 13.7 (7.5/22.3) | 51.5 (43.7/59.2) | 65.0 (40.8/84.6) |
| Sex | Male | 42/82 | 81.0 (65.9/91.4) | 40.0 (24.9/56.7) | 58.6 (44.9/71.4) | 66.7 (44.7/84.4) |  | 47/80 | 95.7 (85.5/99.5) | 18.2 (7.0/35.5) | 62.5 (50.3/73.6) | 75.0 (34.9/(96.8) |
|  | Female | 40/122 | 67.5 (50.9/81.4) | 43.9 (33.0/55.3) | 37.0 (26.0/49.1) | 73.5 (58.9/85.1) |  | 47/109 | 89.4 (76.9/96.5) | 11.3 (4.7/21.9) | 43.3 (33.3/53.7) | 58.3 (27.7/84.8) |
| Aetiology* | Unprovoked | 40/162 | 70.0 (53.5/83.4) | 42.6 (33.7/51.9) | 28.6 (19.9/38.6) | 81.2 (69.5/89.9) |  | 39/134 | 89.7 (75.8/97.1) | 13.7 (7.5/22.3) | 29.9 (21.8/39.1) | 76.5 (50.1/93.2) |
|  | Provoked | 39/161 | 79.5 (63.5/90.7) | 42.6 (33.7/51.9) | 30.7 (21.9/40.7) | 86.7 (75.4/94.1) |  | 54/149 | 94.4 (84.6/98.8) | 13.7 (7.5/22.3) | 38.3 (30.1/47.2) | 81.2 (54.4/96.0) |
| Number of segments with thrombi* | 1 | 49/171 | 59.2 (44.2/73.0) | 42.6 (33.7/51.9) | 29.3 (20.6/39.3) | 72.2 (60.4/82.1) |  | 59/154 | 88.1 (77.1/95.1) | 13.7 (7.5/22.3) | 38.8 (30.5/47.6) | 65.0 (40.8/84.6) |
|  | ≥2 | 33/155 | 97.0 (84.2/99.9) | 42.6 (33.7/51.9) | 31.4 (22.5/41.3) | 98.1 (89.9/100) |  | 35/130 | 100 (85.5/100) | 13.7 (7.5/22.3) | 29.9 (21.8/39.1) | 100 (66.1/100) |
| Pretest probability | Low-to-moderate | 66/181 | 72.7 (60.4/83.0) | 42.6 (33.4/52.2) | 42.1 (32.9/51.7) | 73.1 (60.9/83.2) |  | 77/156 | 93.5 (85.5/97.9) | 13.9 (7.2/23.5) | 51.4 (42.8/60.0) | 68.8 (41.3/89.0) |
|  | High | 16/32 | 81.2 (54.4/96.0) | 42.9 (9.9/81.6) | 76.5 (50.1/93.2) | 50.0 (11.8/88.2) |  | 17/32 | 88.2 (63.6/98.5) | 13.3 (1.7/40.5) | 53.6 (33.9/72.5) | 50.0 (6.8/93.2) |
| Site | Proximal | 41/163 | 90.2 (76.9/97.3) | 42.6 (33.7/51.9) | 34.6 (25.6/44.4) | 92.9 (82.7/98.0) |  | 43/138 | 100 (88.0/100) | 13.7 (7.5/22.3) | 34.4 (26.1/43.4) | 100 (66.1/100) |
|  | Isolated distal | 41/163 | 58.5 (42.1/73.7) | 42.6 (33.7/51.9) | 25.5 (17.1/35.6) | 75.4 (63.5/84.9) |  | 51/146 | 86.3 (73.7/94.3) | 13.7 (7.5/22.3) | 34.9 (26.6/43.9) | 65.0 (40.8/84.6) |

Sensitivity, specificity, positive predictive value (PPV) and negative predictive value (NPV) are provided as relative frequency with according 95% confidence interval for a D-dimer threshold of 0.5mg/L FEU. Absolute frequency (no.) of DVT cases is denoted for all subgroups; *reference group for the stratified analysis of DVT cases by aetiology, site, and number of venous segments with thrombotic material was patients without DVT. Classification of pre-test probability: low-to-moderate, i.e. Wells score 0-2, and high, i.e. Wells score >2. DVT, deep vein thrombosis.

**Table S6. Test characteristics of a fixed D-dimer threshold of 0.25mg/L FEU in subgroups of patients <60 years with**

**suspected DVT according to pretest probability.**

|  |  | **Age <60 years** | | | | |
| --- | --- | --- | --- | --- | --- | --- |
|  |  | No. DVT | Sensitivity [%] | Specificity [%] | PPV [%] | NPV [%] |
| **Total sample** |  |  |  |  |  |  |
| Pretest probability | Low-to-moderate | 71/195 | 91.5 (82.5/96.8) | 16.1 (10.1/23.8) | 38.5 (31.1/46.2) | 76.9 (31.1/46.2) |
|  | High | 21/29 | 90.5 (69.6/98.8) | 25.0 (3.2/65.1) | 76.0 (54.9/90.6) | 50.0 (6.8/93.2) |
| **Outpatients** |  |  |  |  |  |  |
| Pretest probability | Low-to-moderate | 66/181 | 90.9 (81.3/96.6) | 15.7 (9.5/23.6) | 38.2 (30.6/46.3) | 75.0 (53.3/90.2) |
|  | High | 16/23 | 87.5 (61.7/98.4) | 28.6 (3.7/71.0) | 73.7 (48.8/90.9) | 50.0 (6.8/93.2) |

Sensitivity, specificity, positive predictive value (PPV) and negative predictive value (NPV) are provided as relative frequency [%] with according 95% confidence interval.

Classification of pretest probability: low-to-moderate, i.e. Wells score 0-2, and high, i.e. Wells score >2. DVT, deep vein thrombosis.

**Table S7. Diagnostic performance of CRP in patients with suspected DVT by age groups.**

|  |  |  | **Age <60 years** | | | |  |  | **Age ≥60 years** | | | |
| --- | --- | --- | --- | --- | --- | --- | --- | --- | --- | --- | --- | --- |
|  |  | No.  DVT | Sens. [%]  (95%CI) | Spec. [%]  (95%CI) | PPV [%]  (95%CI) | NPV [%]  (95%CI) |  | No.  DVT | Sens. [%]  (95%CI) | Spec. [%]  (95%CI) | PPV [%]  (95%CI) | NPV [%]  (95%CI) |
| Total sample |  | 91/222 | 67.0 (56.4/76.5) | 59.5 (50.6/68.0) | 53.5 (43.9/62.9) | 72.2 (62.8/80.4) |  | 112/221 | 69.6 (60.2/78.0) | 38.5 (29.4/48.3) | 53.8 (45.3/62.1) | 55.3 (43.4/66.7) |
| Sex | Male | 50/99 | 70.0 (55.4/82.1) | 61.2 (46.2/74.8) | 64.8 (50.6/77.3) | 66.7 (51.0/80.0) |  | 62/104 | 71.0 (58.1/81.8) | 33.3 (19.6/49.5) | 61.1 (48.9/72.4) | 43.8 (26.4/62.3) |
|  | Female | 41/123 | 63.4 (46.9/77.9) | 58.5 (47.1/69.3) | 43.3 (30.6/56.8) | 76.2 (63.8/86.0) |  | 50/117 | 68.0 (53.3/80.5) | 41.8 (29.8/54.5) | 46.6 (34.8/58.6) | 63.6 (47.8/77.6) |
| Aetiology† | Unprovoked | 43/174 | 60.5 (44.4/75.0) | 59.5 (50.6/68.0) | 32.9 (22.7/44.4) | 82.1 (72.9/89.2) |  | 45/154 | 64.4 (48.8/78.1) | 38.5 (29.4/48.3) | 30.2 (21.3/40.4) | 72.4 (59.1/83.3) |
|  | Provoked | 45/176 | 73.3 (58.1/85.4) | 59.5 (50.6/68.0) | 38.4 (28.1/49.5) | 86.7 (77.9/92.9) |  | 66/175 | 74.2 (62.0/84.2) | 38.5 (29.4/48.3) | 42.2 (33.1/51.8) | 71.2 (57.9/82.2) |
| Site† | Isolated distal | 45/176 | 46.7 (31.7/62.1) | 59.5 (50.6/68.0) | 28.4 (18.5/40.1) | 76.5 (67.0/84.3) |  | 63/172 | 63.5 (50.4/75.3) | 38.5 (29.4/48.3) | 37.4 (28.2/47.3) | 64.6 (51.8/76.1) |
|  | Proximal | 46/177 | 87.0 (73.7/95.1) | 59.5 (50.6/68.0) | 43.0 (32.8/53.7) | 92.9 (85.1/97.3) |  | 49/158 | 77.6 (63.4/88.2) | 38.5 (29.4/48.3) | 36.2 (27.0/46.1) | 79.2 (65.9/89.2) |
| Number of segments with thrombi† | 1 | 52/183 | 46.2 (32.2/60.5) | 59.5 (50.6/68.0) | 31.2 (21.1/42.7) | 73.6 (64.1/81.7) |  | 71/180 | 64.8 (52.5/75.8) | 38.5 (29.4/48.3) | 40.7 (31.6/50.4) | 62.7 (50.0/74.2) |
|  | ≥2 | 39/170 | 94.9 (82.7/99.4) | 59.5 (50.6/68.0) | 41.1 (30.8/52.0) | 97.5 (91.3/99.7) |  | 41/150 | 78.0 (62.4/89.4) | 38.5 (29.4/48.3) | 32.3 (23.3/42.5) | 82.4 (69.1/91.6) |
| Suspected recurrent DVT |  | 22/42 | 68.2 (45.1/86.1) | 70.0 (45.7/88.1) | 71.4 (47.8/88.7) | 66.7 (43.0/85.4) |  | 39/64 | 56.4 (39.6/72.2) | 52.0 (31.3/72.2) | 64.7 (46.5/80.3) | 43.3 (25.5/62.6) |
| Active cancer |  | 13/25 | 84.6 (54.6/98.1) | 41.7 (15.2/72.3) | 61.1 (35.7/82.7) | 71.4 (29.0/96.3) |  | 30/51 | 83.3 (65.3/94.4) | 33.3 (14.6/57.0) | 64.1 (47.2/78.8) | 58.3 (27.7/84.8) |

Absolute frequency (no.) of DVT cases is denoted for all subgroups; †reference group for the stratified analysis of DVT cases by aetiology, site, and number of venous segments with thrombotic material was patients without DVT. CI confidence interval; DVT, deep vein thrombosis.

**Figure S1. Comparison of the diagnostic performance of D-dimer testing by HemosIL ACL TOP and Siemens Innovance BCS.**


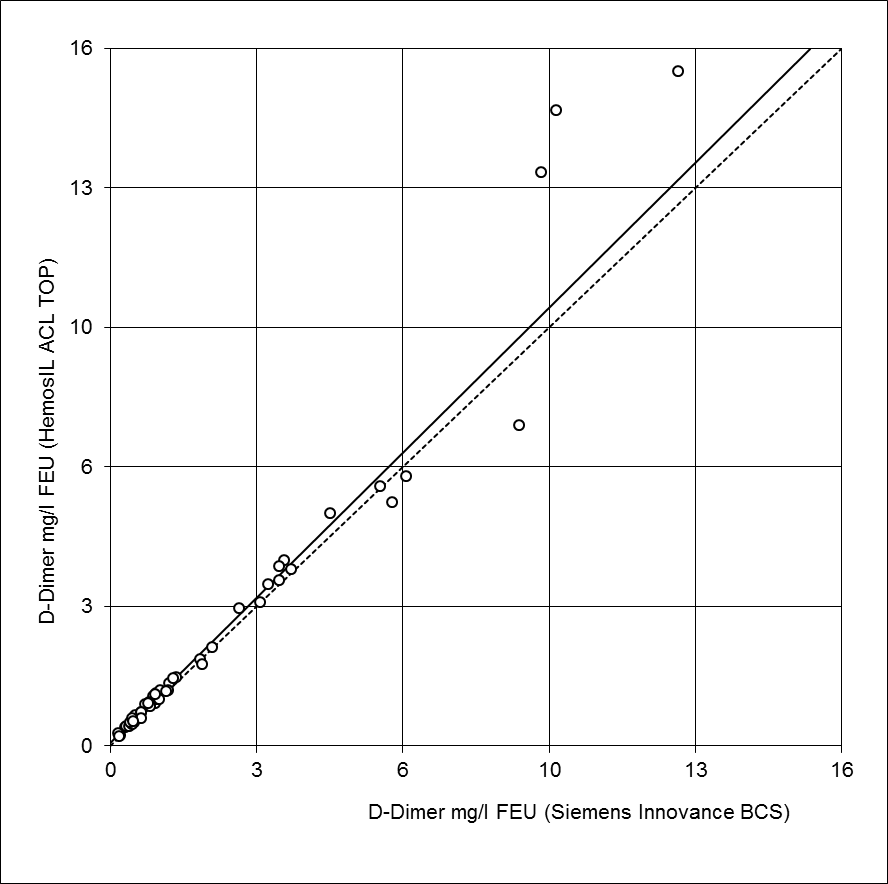


**N=50 individuals per assay**

**r=0.973**

**Figure S2. Flow chart for the optimized threshold of D-dimer for patients <60 years of age.**

**
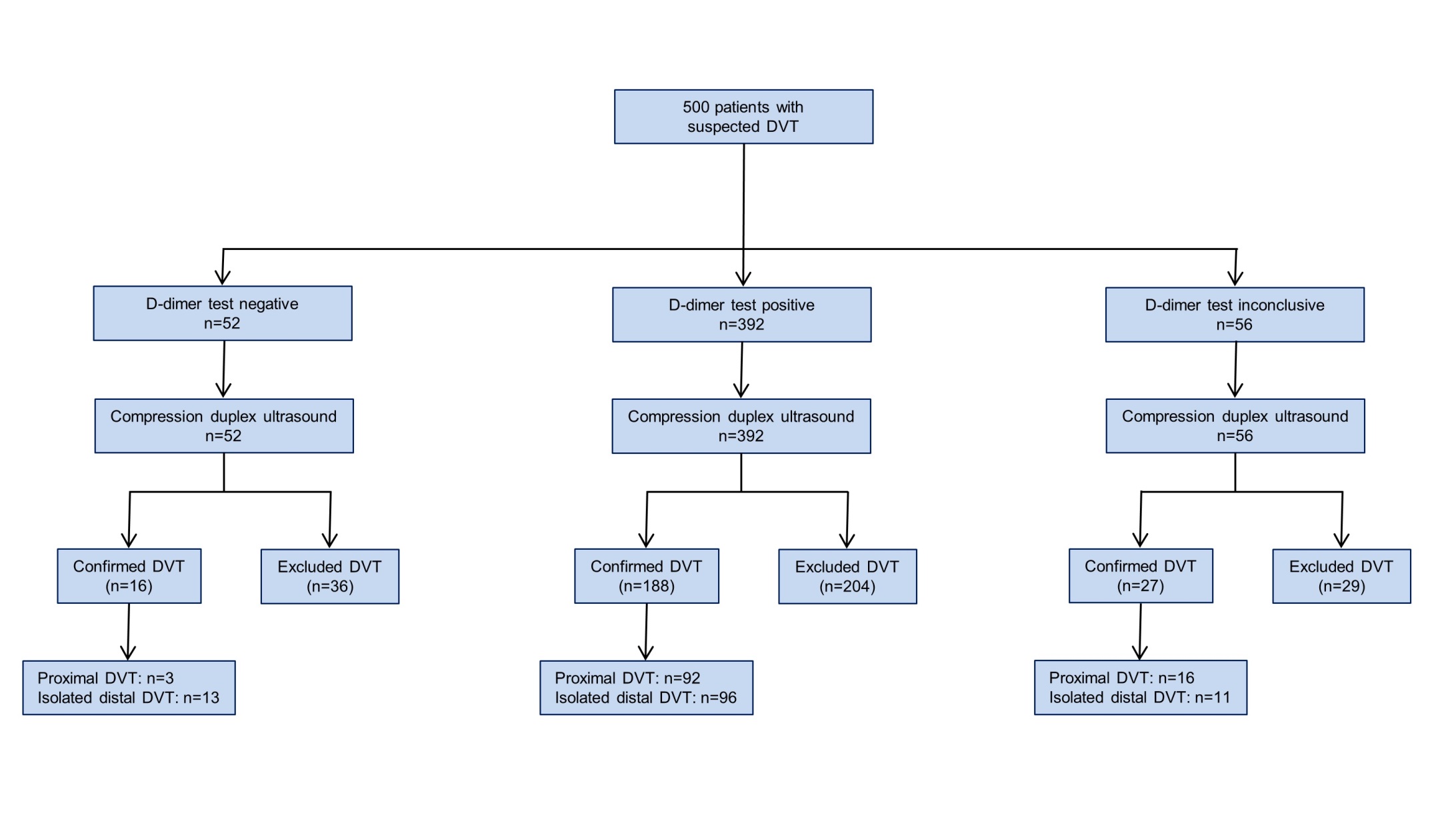
**

Cutoff values of D-dimer were 0.25mg/L FEU for patients <60 years and 0.5mg/L FEU for patients ≥60 years. DVT, deep vein thrombosis.
